# Cost-Effectiveness of Tislelizumab Versus Docetaxel for Previously Treated Advanced Non-Small-Cell Lung Cancer in China

**DOI:** 10.3389/fphar.2022.830380

**Published:** 2022-05-09

**Authors:** Jinhong Gong, Dan Su, Jingjing Shang, Shan Xu, Lidan Tang, Zhiqiang Sun, Guangjun Liu

**Affiliations:** ^1^ Department of Pharmacy, The Affiliated Changzhou NO.2 People’s Hospital of Nanjing Medical University, Changzhou, China; ^2^ Department of Pharmaceutics, College of Pharmaceutical Sciences, Soochow University, Suzhou, China; ^3^ Department of Radiation Oncology, The Affiliated Changzhou NO.2 People’s Hospital of Nanjing Medical University, Changzhou, China

**Keywords:** cost-effectiveness, tislelizumab, partitioned survival model, previously treated, non-small-cell lung cancer

## Abstract

**Background:** Tislelizumab, a new high-affinity programmed cell death protein-1 (PD-1) inhibitor, significantly prolonged the overall survival in pretreated non-small-cell lung cancer (NSCLC). This study aimed to assess the cost-effectiveness of tislelizumab versus docetaxel for this population in China.

**Methods:** A three-state partitioned survival model was developed to simulate advanced NSCLC. Efficacy and safety data were based on a global phase 3 clinical trial (RATIONALE 303). Utilities were mainly extracted from previously published resources. Costs were calculated from the Chinese healthcare system’s perspective, and only direct medical costs were covered. The main outcomes included total costs, life years (LYs), quality-adjusted life years (QALYs), and incremental cost effectiveness ratio (ICER). One-way and probabilistic sensitivity analyses were carried to test the uncertainty of the modeling results. In addition, several scenarios including tislelizumab price before negotiation, different docetaxel price calculation, 50-year time horizon, and alternative utility values were assessed.

**Results:** The model predicted an average gain of 0.62 LYs and 0.51 QALY for tislelizumab vs. docetaxel, at the additional cost of $9,219. The resulting ICER was $15,033.92/LY and $18,122.04/QALY, both below the cost-effective threshold (CET) of three times gross domestic product (GDP) per capita in China. Sensitivity analyses showed that the results are robust over a plausible range for majority of inputs. Utility of progression-free survival (PFS), followed by the price of tislelizumab, had the greatest impact on the ICER. The probability of being cost-effective for tislelizumab was 96.79% at the CET we set.

**Conclusion:** Tislelizumab improves survival, increases QALYs, and can be considered a cost-effective option at current price compared with docetaxel for pretreated advanced NSCLC in China.

## Introduction

Overall, the burden of cancer incidence and mortality is growing rapidly worldwide. According to the latest global cancer statistics GLOBOCAN 2020, lung cancer accounts for the second largest number of cancer around the world as well as ranks the leading cause of cancer-related death (Sung et al., 2021). In China, lung cancer raises a major public health problem with an estimate of 733,300 new cases and 610,200 deaths per year (Chen et al., 2016). The two main classifications of lung cancer are small-cell lung cancer and non-small-cell lung cancer (NSCLC), with the latter being the most common, accounting for 80–85% of all lung cancer diagnoses (Cancer.Net, 2021). NSCLCs, a heterogenous set of diseases, are known to have diverse pathological features. The two predominant NSCLC histological subtypes are adenocarcinoma and squamous cell carcinoma (Chen et al., 2014).

Most NSCLC patients receive systemic therapy, either being diagnosed at an already inoperable stage or experiencing the disease relapse after surgery. Potential clinical benefit of cytotoxic therapy did not look obvious, whereas dramatic breakthrough in the NSCLC management occurred in the first decade of the twentieth century, mainly attributed to targeted therapy against epidermal growth factor receptor (EGFR), BRAF, mesenchymal–epithelial transition factor (MET) mutations, anaplastic lymphoma kinase (ALK), and ROS1 rearrangements (Imyanitov et al., 2021). Most squamous NSCLC and a large portion of non-squamous NSCLC carry no druggable mutations. Immune checkpoint inhibitors (ICIs) through blocking the programmed cell death protein-1 (PD-1) pathway showed dramatic activity in patients with NSCLC. In patients with high expression of PD-L1 (≥50%), front line treatment with pembrolizumab has become the standard of care (CSCO, 2020). Currently, the 5-year survival rate for all people with all types of NSCLC has been significantly improved to about 25% but with only 5–14% and 1% for patients at stage IIIA/B and stage IV, respectively (Allemani et al., 2018; Cancer.Net, 2021). For people with metastatic or recurrent NSCLC that cannot be treated with a targeted therapy, immunotherapy, or immunotherapy plus chemotherapy has been explored and contributed in the spectacular improvement of disease outcomes (Nadal et al., 2019). Clinical trials with second or later line immune checkpoint inhibitors (ICIs) in previously treated advanced NSCLC provide long-term benefit and an acceptable safety profile (Borghaei et al., 2015; Rittmeyer et al., 2017; Wu et al., 2019; Herbst et al., 2021).

Tislelizumab is a humanized monoclonal antibody with high affinity and specificity for PD-1, which was specifically engineered to minimize Fc-γ receptor (FcγR) macrophages binding in order to abrogate antibody-dependent phagocytosis. The low affinity of tislelizumab for the Fc-γ receptor (FcγR) is expected to result in improved anticancer efficacy (Hong et al., 2021). Excitingly, tislelizumab has shown great antineoplastic activity both in hematological cancers and advanced solid tumors (Lee and Keam, 2020). The efficacy and safety of tislelizumab in treating advanced NSCLC have been demonstrated in several clinical studies (Lu et al., 2021; Zhou et al., 2021). Tislelizumab significantly prolongs progression-free survival (PFS) and overall survival (OS) as well as improves the response rate compared with docetaxel in patients with locally advanced or metastatic NSCLC with disease progression after initial platinum-based chemotherapy (Zhou et al., 2021). Currently, due to the exorbitant price of PD-1/PD-L1 antibodies, neither pembrolizumab nor nivolumab present to be cost-effective as second-line treatment for patients with NSCLC in China (Liu et al., 2019; Zhou et al., 2019; Shi et al., 2021). But, the pharmacoeconomic information for tislelizumab in previously treated NSCLC patients is still insufficient in China, and this is required by healthcare decision makers and clinicians to determine the relative role of this novel treatment in NSCLC. Therefore, this study aimed to investigate the cost and effectiveness of tislelizumab versus docetaxel as second- or third-line therapy for patients with locally advanced or metastatic NSCLC from the perspective of the Chinese healthcare system.

## Materials and Methods

### Analytical Overview and Model Structure

A partitioned survival model was developed based on the RATIONALE 303 clinical trial (NCT03358875) to measure the clinical and economic outcomes of two second- or third-line treatment strategies for patients with advanced NSCLC: 1) tislelizumab 200 mg every three weeks (Q3W) and 2) docetaxel 75 mg/m^2^ every three weeks (Q3W) (Zhou et al., 2021). A hypothetical cohort with locally advanced or metastatic squamous or non-squamous NSCLC patients previously treated by one–two prior therapies including at least the platinum-doublet chemotherapy, with negative EGFR mutation or ALK translocation established to compare these two treatments. Patients who suffered disease progression or intolerable adverse events (AEs) were switched to further line treatment. All patients received best supportive care (BSC) during their survival after disease progression on the basis of recent guidelines in China (CSCO, 2020).

**FIGURE 1:**
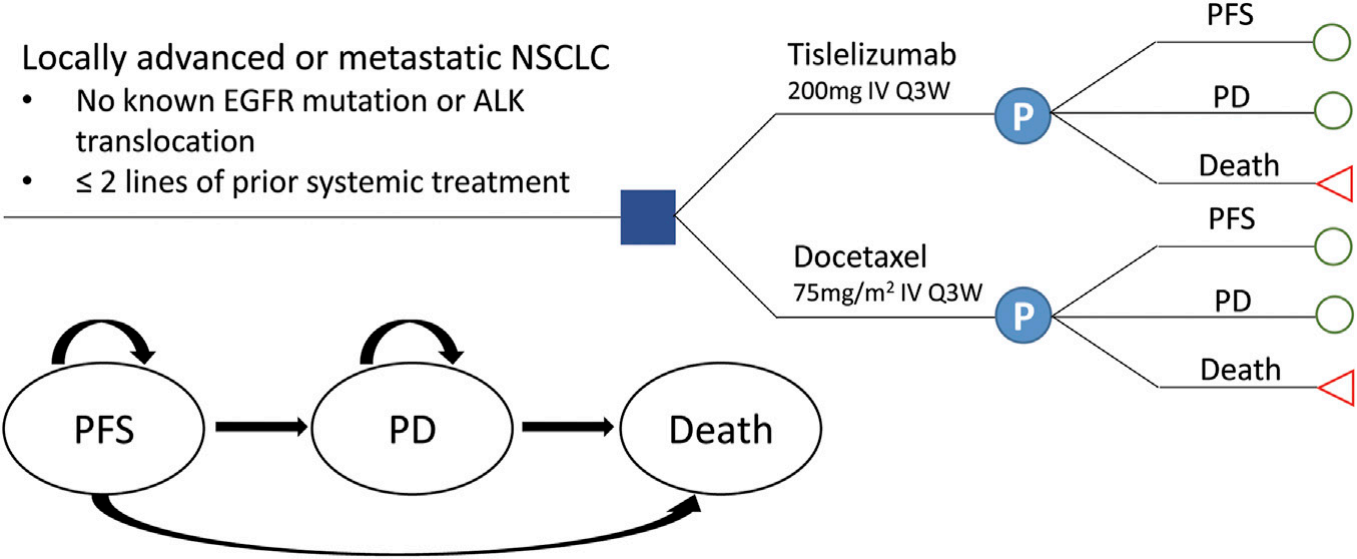
Transition diagram for the three-state partitioned survival model simulating the development of locally advanced or metastatic NSCLC. NSCLC, non-small-cell lung cancer; PFS, progression-free survival; PD, progressive disease.

The model consisted of three mutually exclusive health states: PFS, progressive disease (PD), and death, in accordance with the development of advanced or metastatic NSCLC (Figure 1). All patients were assumed to start in the PFS state, then either stay the same or move to a poorer health state, and cannot go back to the former healthier state. State membership was determined by PFS and OS curves, estimated using parametric models fitted to Kaplan–Meier (K-M) data. The proportion of the cohort in the PFS and death states were determined directly from the PFS and OS data; the proportion of the cohort in the PD state was calculated as OS minus PFS (Shi et al., 2021). The cycle length was set to 3 weeks in line with the tislelizumab and docetaxel schedule. Based on an average life expectancy of 76.34 years in China and the median age of 61.0 years for patients in the RATIONALE 303 study, the time duration was set to 20 years, a period expected to cover the patient’s entire life span. Analyses were performed from the perspective of the Chinese healthcare system, and a 5% discount rate for health utility and cost was assumed (Liu et al., 2020).

### Clinical Data Inputs

K-M survival data for PFS and OS were available from the phase III RATIONALE 303 trial (Zhou et al., 2021). The PFS and OS probabilities were extracted from the PFS and OS K-M curves of each treatment group using GetData Graph Digitizer (version 2.24, http://www.getdata-graph-digitizer.com/download.php). The individual patient data of each K-M curve were reconstructed, and parametric survival analysis was applied to fit the data according to method proposed by Hoyle and Henley, (2011). The long-term clinical outcome was extrapolated using the fitted survival function. The proportional hazard assumption was verified to assess whether independent survival models were to be applied in each treatment arm. Commonly used distributions including exponential, Weibull, Gompertz, log-logistic, log-normal, gamma, and generalized gamma (gengamma) were considered (Ishak et al., 2013). The most appropriate survival function was chosen based on clinical rationality, visual fit, and statistical goodness-of-fit test using Akaike information criteria (AIC) (Akaike, 1974) and Bayesian information criteria (BIC) (Raftery, 1995). The pseudo-individual patient data were fitted using R software (version 4.1.1, http://www.r-project.org).

The log-cumulative hazard plots of both the PFS and OS were not parallel, indicating that the proportional hazards assumptions were not satisfied, and thus two treatment arms were modeled independently (Supplementary Figure S1, S2). Gamma and gengamma distribution functions provided the best fit for OS and PFS data, respectively, in both tislelizumab and docetaxel arms (Supplementary Table S1; Supplementary Figure S3-S6). The median PFS and OS estimates derived from the model simulation were close to those reported in RATIONALE 303 (Supplementary Table S2). The following Excel formulas were used to estimate the survival at different time points (Bennett et al., 2021): 1) gamma distribution: 1-GAMMA.DIST(time, shape, 1/(rate),TRUE); (2) gengamma distribution: IF(Q < 0,GAMMADIST((-Q^-2) * EXP(-Q* -((LN(time)-(mu))/sigma)),-Q^-2,1,1),1-GAMMADIST((-Q^-2) *EXP(-Q*-((LN(time)-(mu))/sigma)),-Q^-2,1,1)). The key clinical inputs are listed in Table 1.

**TABLE 1:** Summary of key parameters input to the model.

Parameter	Baseline value	Range	Distribution	Source
Parametric distribution				
Tislelizumab, PFS, gengamma	mu = 0.9933
Sigma = 1.0262				
Q = −1.0694	—	Fixed in PSA	Estimated	
Docetaxel, PFS, gengamma	mu = 1.0212
Sigma = 0.8465				
Q = −0.0299	—	Fixed in PSA	Estimated	
Tislelizumab, OS, gamma	Rate = 0.05744			
Shape = 1.30351	—	Fixed in PSA	Estimated	
Docetaxel, OS, gamma	Rate = 0.0916			
Shape = 1.3965	—	Fixed in PSA	Estimated	
Tislelizumab: incidence of AEs (%)				
Neutropenia	0.56	0.45–0.67	Beta	Zhou et al. (2021)
Anemia	3.37	2.70–4.04	Beta	Zhou et al. (2021)
Asthenia	1.12	0.90–1.34	Beta	Zhou et al. (2021)
Docetaxel: incidence of AEs (%)				
Neutropenia	27.91	22.33–33.49	Beta	Zhou et al. (2021)
Anemia	6.20	4.96–7.44	Beta	Zhou et al. (2021)
Asthenia	5.43	4.34–6.52	Beta	Zhou et al. (2021)
Utility				
PFS	0.804	0.536–0.840	Beta	Nafees et al. (2017)
PD	0.321	0.031–0.473	Beta	Nafees et al. (2017)
Disutility of AEs				
Neutropenia	0.200	0.149–0.498	Beta	Nafees et al. (2017)
Anemia	0.078	0.078–0.489	Beta	Refer to asthenia
Asthenia	0.078	0.078–0.489	Beta	Nafees et al. (2017)
Drug cost ($)				
Tislelizumab per cycle	674	539–809	Fixed in PSA	NHSA, (2020)
Docetaxel per mg	2.34	1.87–2.80	Gamma	Yaozhi Net, (2021)
Routine follow-up in the PFS state per cycle ($)	55.6	41.7–69.4	Log-normal	Lu et al. (2016)
Subsequent systemic therapy in PD state per cycle ($)	854.1	706.5–992.4	Log-normal	Liu et al. (2019)
BSC per cycle ($)	337.5	158.7–793.7	Log-normal	Lu et al. (2016)
Terminal phase cost ($)	2627.8	2291.8–2966.6	Log-normal	Liu et al. (2019)
AEs cost ($)				
Neutropenia per event	461.5	369.2–553.8	Log-normal	Wu et al. (2012)
Anemia per event	531.7	425.4–638.0	Log-normal	Wu et al. (2012)
Asthenia per event	115.4	92.3–138.5	Log-normal	Wu et al. (2012)
Discount rate (%)	5	0–8	Fixed in PSA	Liu et al. (2020)
Body surface area (m^2^)	1.72	1.38–2.06	Normal	China CDC, (2020)

*PFS*, progression-free survival; *OS*, overall survival; *AEs*, adverse events*; PD*, progressive disease; *BSC*, best supportive care; *PSA*, probabilistic sensitivity analysis.

### Health Utilities

The utilities for patients with NSCLC were based on the Chinese data captured from the study by Nafees et al. (2017), with the base values 0.804 and 0.321 for PFS and PD states, respectively (Table 1). Disutilities associated with grade 3/4 AEs reported in ≥5% of patients in RATIONALE 303 were also included in our model (Table 1). The product of the disutility value of the AE (Nafees et al., 2017) and its incidence rate was used to calculate the utility loss by each AE.

### Cost and Resources Utilization

Costs were estimated from the perspective of the Chinese healthcare system, which only considered direct medical costs (Liu et al., 2020), including drug acquisition, management of grade 3/4 AEs, routine follow-up in the PFS state, subsequent systemic therapy after progression, and BSC and terminal phase treatment (Table 1). In accordance with the RATIONALE 303 trial, 200 mg tislelizumab and 75 mg/m^2^ docetaxel were administered intravenously on the first day of every 3 weeks in two treatment arms, respectively (Zhou et al., 2021). The unit cost of tislelizumab referred to the China’s National Reimbursement Drug List (NRDL) 2020 (NHSA, 2020). Docetaxel has many different manufacturers/specifications in China, and the prices vary widely. Considering the decline trend of exorbitant drug price, especially for anticancer drugs, we selected the most commonly used docetaxel in clinical practice based on the oncologists’ opinions, which was with relatively low and reasonable price. A base-case body surface area (BSA) of 1.72 m^2^ based on an average weight of 65 kg and height of 164 cm (China CDC, 2020) was assumed to calculate the drug cost of chemotherapy (Lu et al., 2018). Grade 3/4 AEs reported in ≥5% of patients in RATIONALE 303 for either tislelizumab or docetaxel were included. The costs related to AEs were calculated by multiplying the incidence of AEs by the managing costs per event. Based on the clinical practices, decreased neutrophil count and decreased white blood cell count are moderate AEs compared to neutropenia and leukopenia, which generally do not require additional treatment, and the treatment for neutropenia also treats leukopenia. Therefore, the costs of those AEs were excluded from the total costs. Costs for AEs and others were all derived from previously published studies (Wu et al., 2012; Lu et al., 2016; Liu et al., 2019). All costs were reported in 2021 United States dollars (average rate for the first half of 2021: $1 = CNY 6.4698) (China Foreign Exchange Trade System, 2021).

### Base-Case Analysis

The primary outcomes included total costs, life years (LYs), and quality-adjusted life years (QALYs). The incremental cost-effectiveness ratio (ICER) was also calculated and compared with a cost-effective threshold (CET) of $32872 per QALY, equal to three times China’s gross domestic product (GDP) per capita in 2019 (Liu et al., 2020).

### Sensitivity Analysis

A series of sensitivity analyses, including one-way and probabilistic sensitivity analyses (PSA), were carried out to evaluate the robustness of the model and uncertainty incurred from parameter estimation. In one-way sensitivity analysis, all variables varied across a plausible range which were based on 95% confidence intervals (95% CIs) or by assuming a variance of ±20%. In addition, the lower and upper bound of utility and disutility of AEs were derived from the minimum and maximum among different countries, respectively (Nafees et al., 2008) (Table 1). A second-order Monte Carlo simulation with 10,000 iterations was performed for PSA. All parameters simultaneously varied with assumed statistical distributions (Briggs et al., 2012). Incidence and health utility used the beta distribution, cost and medical resource utilization parameters used the log-normal distribution, and the price of docetaxel used the gamma distribution (Table 1).

### Scenario Analysis

Several scenarios were considered to explore the potential impact on the model result. Including the tislelizumab price before China’s national negotiation, docetaxel price was estimated according to the average prices of pharmaceutical purchases in various provinces across the country (Shi et al., 2021), time horizon of 50 years, PFS utility value without considering AEs disutility, and utility values frequently cited in NSCLC-related cost-effectiveness studies (Nafees et al., 2008).

All analyses were performed in TreeAge Pro 2020 (TreeAge Software, Inc., Williamstown, MA).

## Results

### Base-Case Analysis

The estimated mean PFS duration and total life expectancy for patients receiving tislelizumab were 0.95 LYs and 1.88 LYs, respectively, with an increase of 0.62 LYs compared with those for patients receiving docetaxel. Incorporating the quality of life, tislelizumab produced a marginal 0.51 QALYs (1.06 vs 0.55) with an incremental cost of $9,219 (33,835 vs 24,616), which includes the additional cost of $9,153 in the PFS state. The model indicated that the ICER per LY and per QALY for tislelizumab versus docetaxel were $15033.92 and $18122.04, respectively, far below the CET of $32872/QALY. The benefit associated with tislelizumab mainly depended on the prolongation of PFS, thereby incurring the large proportion of incremental cost in this state (Table 2).

**TABLE 2:** Base-case analysis results.

Item	Tislelizumab	Docetaxel	Difference
Mean LYs			
PFS	0.95	0.33	0.62
PD	0.93	0.93	0
Total	1.88	1.26	0.62
Mean QALYs			
PFS	0.76	0.25	0.51
PD	0.30	0.30	0
Total	1.06	0.55	0.51
Cost ($)			
PFS	12,177	3,024	9,153
PD	19,051	18,978	613
Dead	2, 607	2,614	−7
Total	33,835	24,616	9,219
ICER ($/LY)	15,033.92^a^		
ICER ($/QALY)	18,122.04^a^		

*PFS*, progression-free survival; *PD*, progressive disease; *LYs*, life years; *QALYs,* quality-adjusted life years; *ICER*, incremental cost-effectiveness ratio.

a Compared with docetaxel.

### Sensitivity Analyses

**FIGURE 2:**
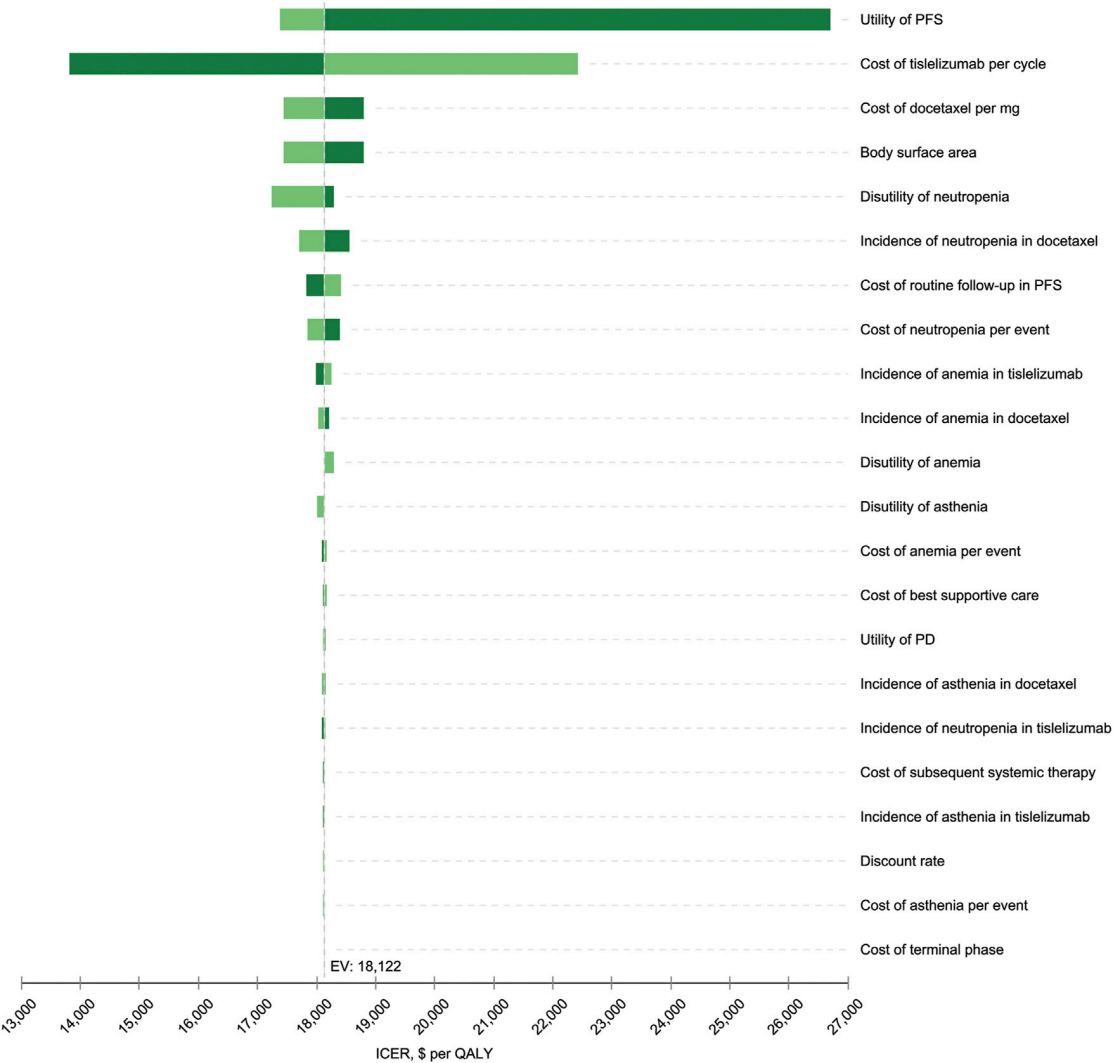
Tornado diagram for the one-way sensitivity analysis. The variables are listed in descending order by their influence on the ICER. The dashed line intersecting the bars represents the ICER of $18,122 per QALY from the base-case result. PFS, progression-free survival; PD, progressive disease; EV, estimated value; ICER, incremental cost-effectiveness ratio; QALY, quality-adjusted life year.

**FIGURE 3:**
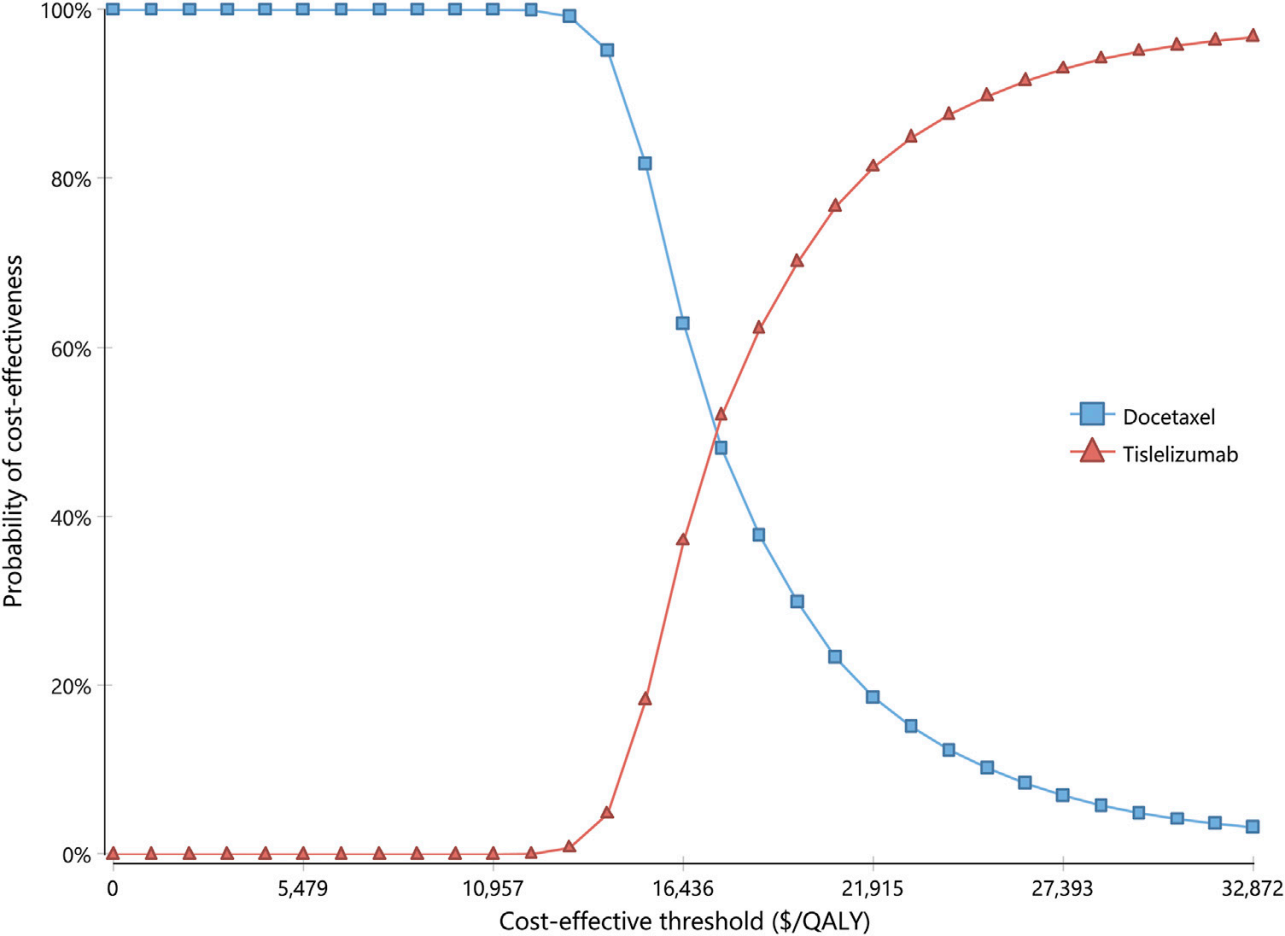
Cost-effectiveness acceptability curve for probabilistic sensitivity analysis. The curve shows the probability of being cost-effective at different CETs by using tislelizumab and docetaxel. CETs, cost-effective thresholds; QALY, quality-adjusted life year.

Results for one-way sensitivity analysis were illustrated by a tornado diagram (Figure 2). The utility of PFS and cost of tislelizumab were the two main influential parameters on the ICER, accounting for about 96.2% in total. Several other parameters, including cost of docetaxel, BSA, disutility of neutropenia, incidence of neutropenia in the docetaxel arm, cost of routine follow-up in the PFS state, and cost of neutropenia, had a slight effect on ICER. All the varied ICERs did not exceed the threshold of three times GDP per capita. After running 10,000 iterations of Monte Carlo simulation, the mean costs for tislelizumab and docetaxel arms were 33,835 (SD ± 262) and 24,613 (SD ± 573), with mean QALYs of 1.06 (SD ± 0.16) and 0.55 (SD ± 0.08), respectively. Figure 3 presented the results of PSA using the cost-effectiveness acceptability curve (CEAC). The probability of tislelizumab being cost effective in comparison to docetaxel was 96.79% at the CET of $32872/QALY. In addition, the probabilities of tislelizumab being cost-effective were 93.02, 81.39, 37.12, and 0% at the CET of 2.5, 2, 1.5, and 1 times GDP per capital, respectively.

### Scenario Analyses

The results of scenario analyses are displayed in Table 3. When the tislelizumab price before China’s national negotiation was adopted, its ICER versus docetaxel would be far beyond the CET of three times GDP per capita and could not be cost-effective for second- or third-line NSCLC treatment. On the contrary, when the docetaxel price was derived from a recently published study which calculated the average prices of pharmaceutical purchases in various provinces across the country (Shi et al., 2021), the ICER would dramatically decrease to $6068, even much lower than one time GDP per capita ($10957). Expanding the time horizon to 50 years or using the PFS utility value without subtracting, AE-related disutility caused little impact on the ICER. When using the utility values of 0.673 and 0.473 for PFS and PD, respectively (Nafees et al., 2008), the ICER presented some extent of an increasing trend but still not exceeding the CET we set.

**TABLE 3:** Scenario analyses results.

Scenario	ICER ($/QALY)
Tislelizumab cost before negotiation	101,904.40
Docetaxel cost based on Shi et al. (2021)	6068.40
Time horizon of 50 years	18,122.04
PFS utility value without considering AE disutility	18,764.37
Utility based on Nafees et al. (2008)	22,377.28

*PFS*, progression-free survival; *PD*, progressive disease; AEs, adverse events; *ICER*, incremental cost-effectiveness ratio; *QALY,* quality-adjusted life year.

## Discussion

Based on our information, this study first assessed the cost and effective value of tislelizumab versus docetaxel at the second- or third-line setting for the treatment of NSCLC. In our analysis, tislelizumab was estimated at an ICER of $18122.04 per QALY compared with docetaxel, which was below the pre-set CET of three times GDP per capita, and lay within 1.5 and 2.0 times GDP per capita.

As a leading cause of cancer death, NSCLC takes a key position in life expectancy improvements compared to other tumor types (Howlader et al., 2020). The novel immunotherapy with ICIs, such as nivolumab, pembrolizumab, atezolizumab, and durvalumab, has been evolving and showed promising clinical outcomes in patients with NSCLC (Nadal et al., 2019). Meanwhile, the high price of new anticancer drugs virtually exposes both patients and the society to critical economic burden. Therefore, it is essential to weigh between the clinical efficacy and escalating medical expenditure. Several economic studies around the world assessed the ICIs as second-line treatments for NSCLC, and the cost-effectiveness results were inconsistent among different countries. A total of three studies, one each from Switzerland (Matter-Walstra et al., 2016), Australia (Gao and Li, 2019), and England (Rothwell et al., 2021) showed that nivolumab is not cost-effective compared with docetaxel for patients with previously treated advanced NSCLC except in England. One US-based study found that pembrolizumab is cost-effective versus docetaxel for second-line treatment of advanced NSCLC patients with PD-L1 ≥50% (Huang et al., 2017). However, neither nivolumab nor pembrolizumab was proved to be a cost-effective therapeutic option for pre-treated NSCLC in China. The ICERs for nivolumab and pemborlizumab versus docetaxel were $93,307 and $107,846, respectively, and both were higher than the threshold of three times GDP per capita in China (Liu et al., 2019; Shi et al., 2021).

One factor influencing our model the most was the health utility in PFS, and this was in accordance with studies on pembrolizumab and nivolumab, in which it ranked the second and third, respectively (Liu et al., 2019; Shi et al., 2021). In this study, we extracted the Chinese health utility data from an international study, which measured the health utilities for metastatic NSCLC through a time trade-off interview of oncologists from different European and Asian countries (Nafees et al., 2017). The literature cited the most was a UK-based publication also authored by Nafees et al (2008), which evaluated utility values in patients with NSCLC receiving second-line chemotherapies ( Qiao et al., 2021). In this scenario, the ICER of tislelizumab vs. docetaxel was $22377.28, slightly higher than the base-case result but still did not exceed our CET.

The price of tislelizumab was the second sensitive factor, resulting an ICER of $13821 to $22423 per QALY as the cost of tislelizumab per cycle increased from $539 to $674. In addition, scenario analysis indicated that tislelizumab was not cost-effective at the price before China’s national negotiation in 2020. The costs of nivolumab and pembrolizumab were also observed to be the main factors affecting the results of ICERs (Liu et al., 2019; Shi et al., 2021). Obviously, the drug-pricing negotiations sponsored by the China’s National Healthcare Security Administration (NHSA) play a great positive role in controlling medical expenses incurred by high-cost oncology drugs.

The threshold of once and three times GDP per capita for each QALY recommended by the World Health Organization (WHO) is commonly cited in health economic evaluations (Robinson et al., 2017; Ochalek et al., 2020). However, it has also been argued to be not always applicable in all countries. Actually, the CETs in most cases were not unique and varied among different countries/regions as well as different disease situations (Laupacis et al., 1992; Charlton and Rid, 2019; Ochalek et al., 2020; Vanness et al., 2020). Explorations of appropriate CET for health technology assessment (HTA) are limited still less than the disease-specific CET. Ochalek et al (2020) estimated that the CET in China was below one time GDP per capita using a marginal productivity approach. Another study based on the value of the statistical life (VSL) approach found that the CET per QALY in China is close to 1.5 times (ranging from 1.2 to 3.0) of GDP per capita (Cai et al., 2021). Nevertheless, it was still proposed that a higher threshold should be afforded for serious diseases with a high mortality risk (Cai et al., 2021; Lakdawalla and Phelps, 2021). Three times of GDP per capita is the widely used criterion to judge cost-effectiveness by scholars and policymakers in China, especially in the field of cancer treatment (Liu et al., 2019; Liu et al., 2020; Gong et al., 2021; Shi et al., 2021). Based on the aforementioned considerations, a CET of three times GDP per capita might be acceptable in the context of the advanced/metastatic NSCLC in China. Our study showed that the ICER of tislelizumab versus docetaxel for NSCLC was $18122.04, which was below the threshold of three times GDP per capita, so the incremental cost incurred by tislelizumab was considered acceptable. PSA revealed that the probability of tislelizumab being cost-effective was 37.12% at the CET of 1.5 times GDP per capita.

A long enough time horizon to accommodate life expectancy for advanced NSCLC patients is recommended to reflect important differences in costs and outcomes between the treatments being compared (NICE, 2013). For the base-case analysis, a 20-year time horizon was used and was varied in sensitivity analyses. The median follow-up available from RATIONALE 303 was 19 months (Zhou et al., 2021). The lifetime horizon required extrapolating the survival data many years beyond the trial duration. The base-case OS modeling approach projected that 0% of patients would still be alive at 20 years after initiating tislelizumab, therefore justifying the use of a 20-year time horizon. Accordingly, the scenario analysis of 50-year yielded the same ICER as 20 years.

Several limitations of the current analysis should be mentioned. First, although RATIONALE 303 is a multicenter randomized phase 3 trial intended to provide comprehensive clinical evidence for tislelizumab in NSCLC treatment, it could not completely reflect the real world data. Second, the long-term survival outcomes were based on extrapolation of K-M curves through parametric survival fitting, even though statistical and internal validation were implemented; this will inevitably cause some biases and affect our modeling results. It will be necessary to update this study when long-term survival data are reported. Third, the costs of the healthcare were mainly derived from the previous literature. However, sensitivity analyses by varying the costs with a wide range revealed cost-related parameters, except tislelizumab and docetaxel prices, had little impact on the economic outcomes. The costs for tislelizumab and docetaxel were calculated based on the latest prices in 2021 which could represent the real world data more accurately.

## Conclusions

Tislelizumab has been proved to significantly improve the progression-free survival and overall survival for previously treated patients with advanced NSCLC despite the level of PD-L1 expression. The results of the present study suggest that tislelizumab increases the quality-adjusted expectancy at an acceptable incremental costs. Hence, tislelizumab is able to be considered a cost-effective strategy compared with docetaxel in these advanced NSCLC patients in China.

## Data Availability

The original contributions presented in the study are included in the article/Supplementary Material, further inquiries can be directed to the corresponding authors.
