# Supplementary material for: Cost-Effectiveness of Tislelizumab Versus Docetaxel for Previously Treated Advanced Non-Small-Cell Lung Cancer in China
Source: Front Pharmacol. 2022 May 9;13:830380. doi: 10.3389/fphar.2022.830380 (PMC9124929; doi:10.3389/fphar.2022.830380)
Supplement: Supplementary file 1 [file DataSheet1.DOCX]

**Supplementary Materials**

**TABLE S1.** Statistical fits of OS and PFS curves

| **Item** | **AIC** | | **BIC** | |
| --- | --- | --- | --- | --- |
|  | **Tislelizumab** | **Docetaxel** | **Tislelizumab** | **Docetaxel** |
| OS | | | | |
| Exponential | 2585.09 | 1364.63 | 2589.39 | 1368.21 |
| Weibull | 2574.33 | 1356.96 | 2582.92 | 1364.13 |
| Gompertz | 4613.90 | 2604.00 | 4622.49 | 2611.17 |
| Log-logistic | 2657.00 | 1400.34 | 2665.59 | 1407.51 |
| Log-normal | 2590.22 | 1358.16 | 2598.81 | 1365.32 |
| Gamma | 2574.41 | 1355.69 | 2583.00 | 1362.86 |
| Gengamma | 2576.31 | 1356.58 | 2589.20 | 1367.33 |
| PFS | | | | |
| Exponential | 2820.57 | 1092.59 | 2824.84 | 1096.16 |
| Weibull | 2818.04 | 1076.08 | 2826.59 | 1083.22 |
| Gompertz | - | - | - | - |
| Log-logistic | 2873.61 | 1124.28 | 2882.16 | 1131.42 |
| Log-normal | 2739.78 | 1057.77 | 2748.33 | 1064.91 |
| Gamma | 2822.25 | 1069.42 | 2830.80 | 1076.57 |
| Gengamma | 2709.37 | 1059.75 | 2722.20 | 1070.47 |

*OS*, overall survival; *PFS*, progression-free survival; *AIC*, Akaike Information Criterion; *BIC,* Bayesian Information Criterion.

**TABLE S2.** Internal validation of selected model

| **Item** | **Clinical data** | **Model** |
| --- | --- | --- |
| Median OS of tislelizumab (months) | 17.2^*^ (95% CI 15.28, 20.04) | 17.2 |
| Median OS of docetaxel (months) | 11.9 (95% CI 10.18, 13.93) | 11.8 |
| Median PFS of tislelizumab (months) | 4.1^*^ (95% CI 3.75, 5.03) | 4.1 |
| Median PFS of docetaxel (months) | 2.6 (95% CI 2.17, 3.78) | 2.8 |
| the 12-month PFS rate in tislelizumab | 23.3% | 22.0% |
| the 12-month PFS rate in docetaxel | 5.7% | 4.4% |

*OS,* overall survival; *PFS,* progression-free survival; ^*^ p<0.001.

**FIGURE S1.** Log-cumulative hazard plots of PFS data

**FIGURE S2.** Log-cumulative hazard plots of OS data

**FIGURE S3.** Tislelizumab PFS K-M curve fitting and extrapolation

**FIGURE S4.** Docetaxel PFS K-M curve fitting and extrapolation

**FIGURE S5.** Tislelizumab OS K-M curve fitting and extrapolation

**FIGURE S6.** Docetaxel OS K-M curve fitting and extrapolation


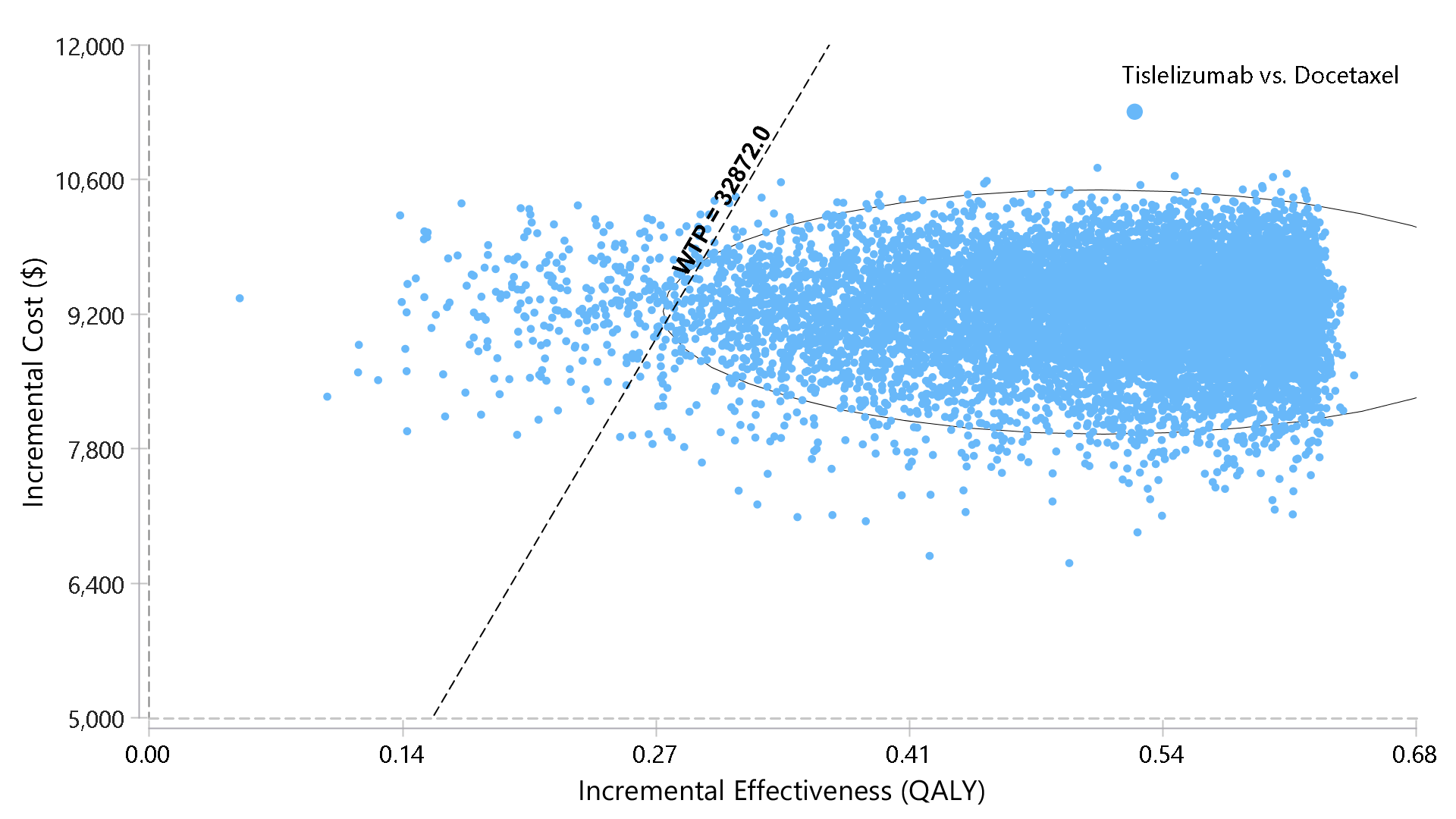


**FIGURE S7.** Scatter plot for incremental cost-effectiveness of tislelizumab vs. docetaxel


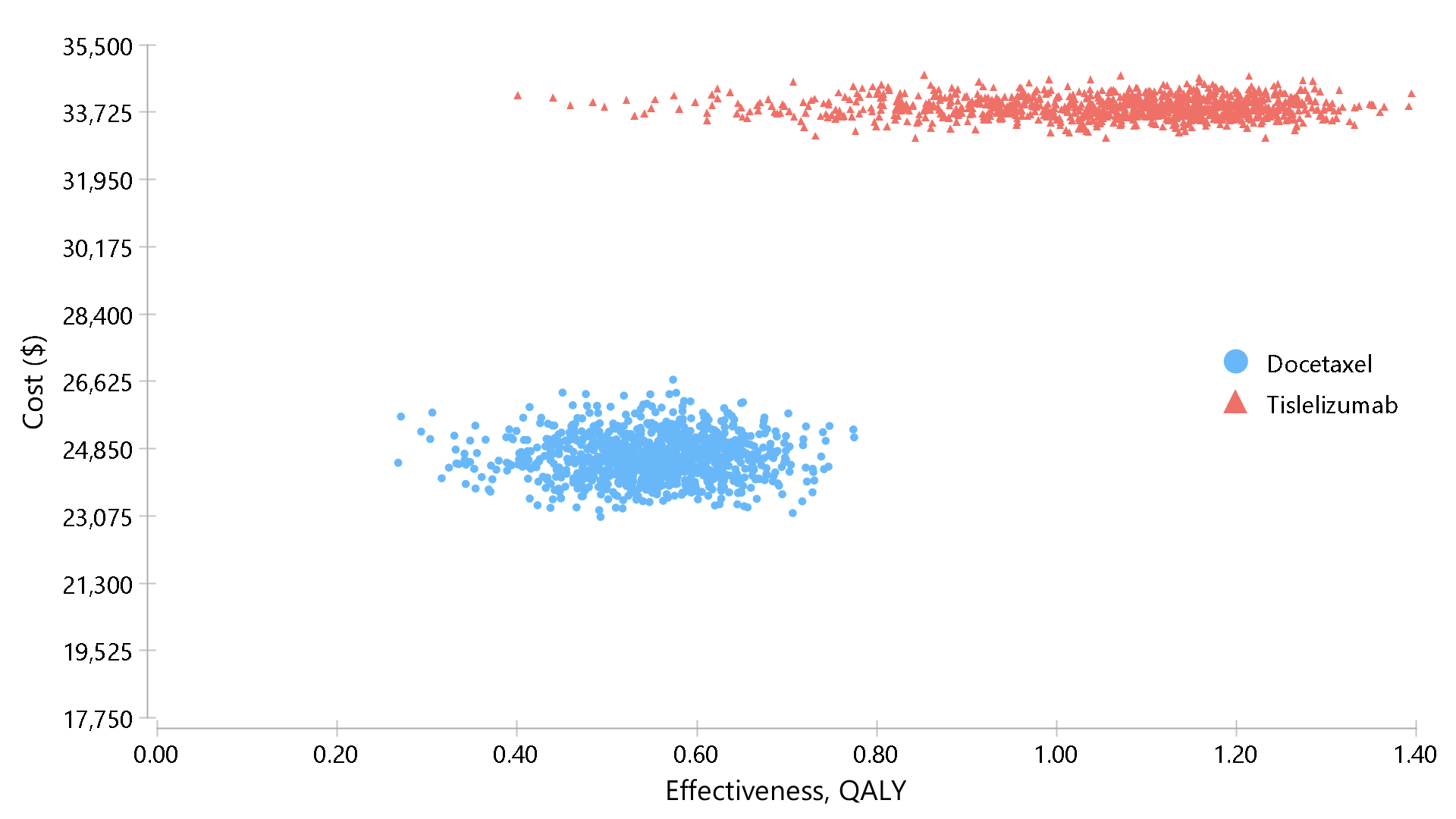


**FIGURE S8.** Scatter plot for cost-effectiveness of tislelizumab vs. docetaxel
